# A case of massive hemoptysis caused by lung cancer saved by V-V ECMO and hemostasis achieved by radiotherapy

**DOI:** 10.1007/s13691-023-00637-3

**Published:** 2023-11-02

**Authors:** Daichi Takizawa, Toshiki Ishida, Hidehiko Nakano, Hiroaki Tachi, Yusuke Yamamoto, Kei Shimizu, Takashi Iizumi, Taisuke Sumiya, Kayoko Ohnishi, Hideyuki Sakurai

**Affiliations:** 1 Department of Radiation Oncology, Hitachi General Hospital, 2-1-1 Jonantyo, Hitachi, Ibaraki 317-0077 Japan; 2 Department of Radiation Oncology, Faculty of Medicine, University of Tsukuba, 1-1-1 Tennodai, Tsukuba, Ibaraki 305-8577 Japan; 3 Department of Emergency and Critical Care Medicine, Hitachi General Hospital, 2-1-1 Jonantyo, Hitachi, Ibaraki 317-0077 Japan; 4 Department of Respiratory Medicine, Hitachi General Hospital, 2-1-1 Jonantyo, Hitachi, Ibaraki 317-0077 Japan; 5 Department of Radiology, School of Medicine, International University of Health and Welfare, 4-3 Kozunomori, Narita, Chiba 286-8686 Japan

**Keywords:** Massive hemoptysis, Oncology emergency, V-V ECMO, Radiotherapy

## Abstract

Massive hemoptysis is one of the fatal complications of lung cancer. There is no established standard treatment method for it, and it often causes sudden suffocation, and some cases may be difficult to save. A 63-year-old man was admitted to the hospital with dyspnea, and developed massive hemoptysis from lung cancer shortly after admission. The tumor had obstructed the right main bronchus and had invaded the right pulmonary artery. Surgery and interventional radiology were judged impossible. The patient was successfully saved by the introduction of Veno-Venous Extra Corporeal Membrane Oxygenation (V-V ECMO), and hemostasis was obtained by radiotherapy. Two months after completion of radiotherapy, he was weaned off the ventilator and discharged on his own. He died of increased peritoneal dissemination and other complications 1 year and 1 month later, but no recurrence of hemoptysis was noted until his death. We experienced a case of massive hemoptysis in which V-V ECMO and radiation therapy succeeded in saving life and stopping bleeding. The use of V-V ECMO by emergency care teams and multimodality therapy, including radiotherapy, were effective for massive hemoptysis from lung cancer.

## Introduction

The treatment of massive hemoptysis from lung cancer has not been established in the guidelines, but it is known that 5–10% of non-small cell lung cancers are complicated by severe hemoptysis [1], and some cases are difficult to treat. In particular, it has been reported that bleeding from the pulmonary artery has a higher fatality rate than bleeding from the bronchial artery, and the major cause of death in massive hemoptysis is suffocation [2]. Radiotherapy is effective for hemostasis, but it is known to have a weakness in that it takes a long time for the therapeutic effect to appear. The hemostasis mechanism of tumor bleeding due to radiotherapy is explained by increased adhesion of platelets to the vascular endothelium after a few fractions of radiotherapy and by causing vessel fibrosis combined with tumor remission in the long term [3].

We report a case of oncology emergency by massive hemoptysis from primary lung cancer with low pulmonary function and extensive invasion of the right pulmonary artery, in which neither surgery nor Interventional Radiology (IVR) is indicated, successfully saved by respiratory management with Veno-Venous Extracorporeal Membrane Oxygenation (V-V ECMO) and radiotherapy. There have been no previous reports of successful treatment of massive hemoptysis with ECMO and radiotherapy. The patient survived the acute phase with the use of V-V ECMO and a ventilator, and after hemostasis and tumor shrinkage due to radiotherapy.

## Case

**Fig. 1:**
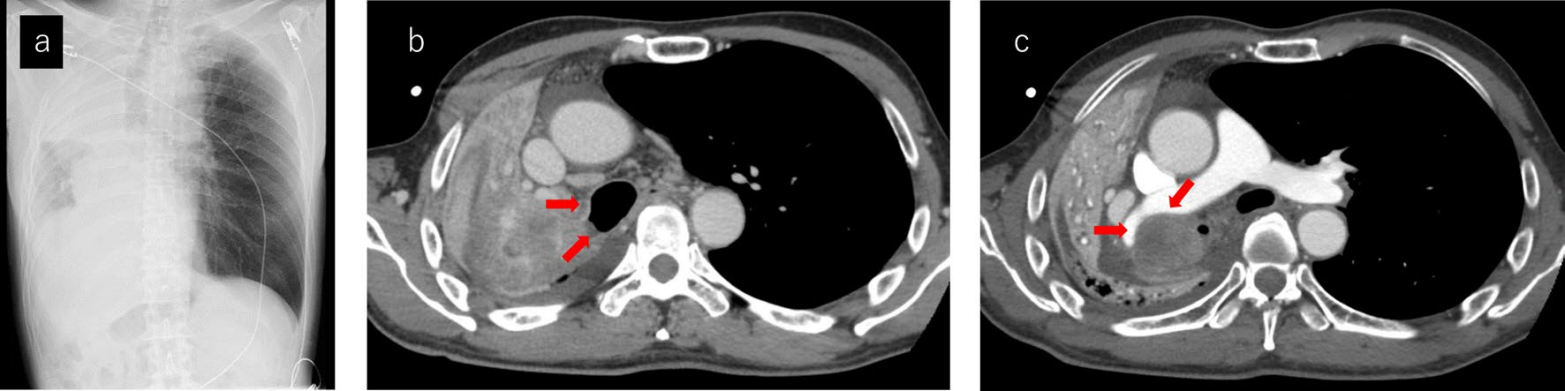
**a**–**c** Chest X-ray and CT at the first admission. **a** Atelectasis of the right lung. **b** Right main bronchus invasion (red arrows). **c** Right pulmonary artery invasion (red arrows)

A 63-year-old man presented to the emergency department by ambulance with sudden right chest pain and dyspnea. In the emergency department, he had 60% O_2_ pulse oximetry on room air and started using 10 L of reservoir mask oxygen. A chest X-ray (Fig. 1a) showed obstruction of the right bronchus and extensive atelectasis in the right lung, and chest CT (Fig. 1b, c) showed that there was infiltration into the right main bronchi and the right pulmonary artery. On the day of admission, ampicillin/sulbactam were started for obstructive pneumonia, and he was put on NPPV. At night, AF tachycardia and hypotension thought to be derived from sepsis were observed, and since the systolic blood pressure had fallen to the 70s, continuous administration of landiolol 15 mg/h and norepinephrine 0.3*γ* was started. Tracheal intubation was performed the next day.

**Fig. 2:**
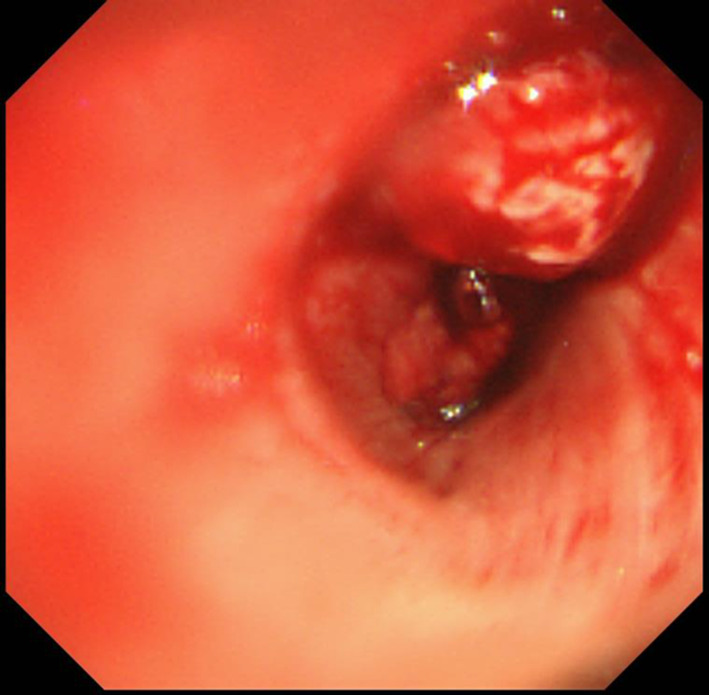
Bronchoscopy showed bleeding from a tumor in the right main bronchus

On the third day of hospitalization, massive hemoptysis was observed, and blood flowed into the unaffected lung, leading to severe respiratory failure. Bronchoscopy revealed hemorrhage and a protruding mass from the right upper lobe to the right main bronchus (Fig. 2). A biopsy was impossible due to bleeding, but tissue adhered when the intubation tube was replaced, and the patient was diagnosed with primary lung adenocarcinoma of clinical stage T2bN3M0 (Stage IIIB, UICC 8th) after the examinations of whole-body and head contrast-enhanced CT. And as a result of pathological diagnosis, programmed death ligand 1 expression was high (Tumor Proportion Score > 95%). Since it was difficult to maintain oxygenation and cardiac arrest was imminent, V-V ECMO was first urgently introduced, and separated lung ventilation was performed to protect the unaffected lung, and deep sedation was also started. V-V ECMO was initiated with femoral extraction and jugular return. As a result of the multidisciplinary joint conference, due to low lung function and malnutrition, surgery was not indicated. And, IVR was not indicated due to bleeding from the tumor invading the pulmonary artery. Radiation therapy aimed at hemostasis and improvement of right atelectasis takes long time, but it was judged possible even under ventilator control. Fortunately, after the introduction of V-V ECMO, partial improvement in respiratory condition was obtained. After switching the intubation method to tracheostomy, it was decided to start radiotherapy. A tracheostomy was performed while V-V ECMO was in use, and a 7.5 mm endotracheal intubation tube was inserted into the left bronchus to continue isolated lung ventilation for the purpose of protecting the unaffected lung, and V-V ECMO was withdrawn. The total amount of transfusion used from the first day to the ninth day of hospitalization was 16 units of RBC, 24 units of FFP, and 50 units of PC.

**Fig. 3:**
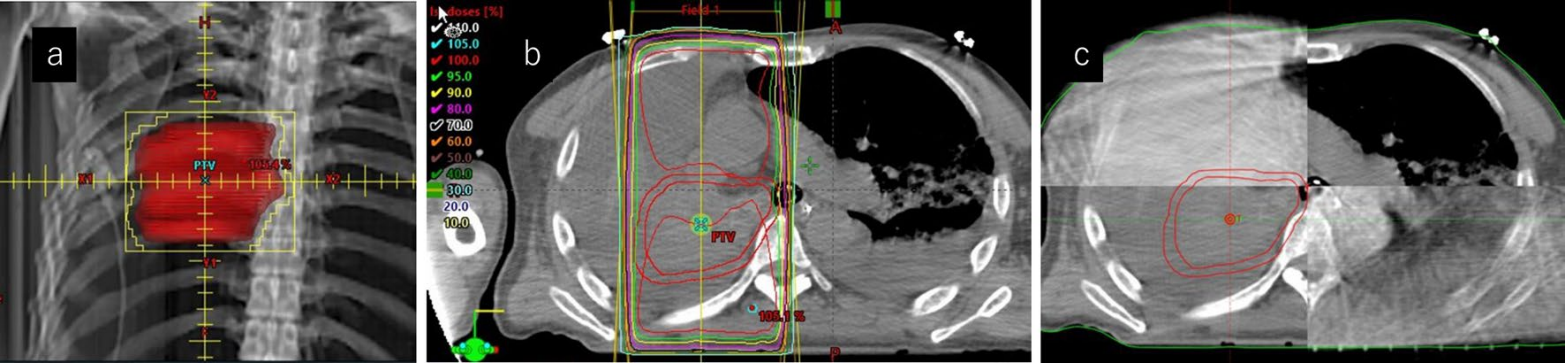
**a**–**c** Radiotherapy. **a** Planning target volume is red contouring area. **b** 3D-CRT planning. **c** Cone beam CT

Radiotherapy was then started at 30 Gy/10 fr. Three-dimensional conformal radiation therapy (3D-CRT) was used as the irradiation method. The purpose of irradiation was to stop bleeding and improve atelectasis in the right lung, and Clinical Target Volume (CTV) was focused only on the primary tumor (Fig. 3a, b). Because the position of the tumor shifted due to increased pleural effusion and atelectasis, Image-Guided Radiotherapy (IGRT) was used for each treatment, and position correction was performed using cone beam CT (Fig. 3c). Each irradiation was carried out under strong sedation as a countermeasure against body movement, and emergency department staff and a clinical engineer were present. From the start until about the third day after the start of irradiation, the patient continued to produce approximately 5 ml of bloody sputum per hour. Thereafter, the amount of bloody sputum gradually decreased, and by about the fifth day after the start of irradiation, it had changed to a normal yellow sputum. At the end of radiotherapy, almost no bloody sputum was drawn even with aspiration.

**Fig. 4:**
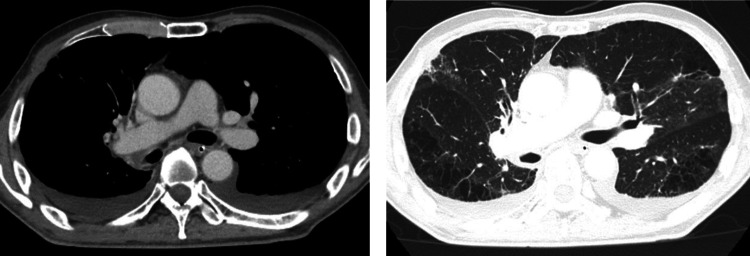
Chest CT 1 month after radiotherapy showed that atelectasis in right lung improved and the primary lung cancer shrank

One week after the end of radiotherapy, the atelectasis in the right lung improved to some extent. In addition, thromboembolism of both pulmonary arteries was observed, and heparin was started, but recurrence of airway bleeding was not observed. About 1 month after the end of radiotherapy, the patient was completely weaned from the ventilator, and CT showed a reduction in the primary tumor of lung cancer and an improvement in air content in both lungs (Fig. 4). The speech cannula was removed about 2 months after the end of radiotherapy, and he was discharged ambulatory. The lung cancer treatment was continued on an outpatient basis, and additional irradiation of 30 Gy/10 fr to the shrinked primary tumor followed by treatment with pembrolizumab. The patient died of cancer 1 year and 1 month after the initial hospitalization due to an increase in peritoneal dissemination and associated complications of ileus. During the follow-up, recurrence of hemoptysis was not observed until death.

## Discussion

There is no clear definition of massive hemoptysis, but there is a report that defines it as a bleeding amount of 100 ml or more per day to 1000 ml over several days [4], and the mortality rate is particularly high in cases of rapid hemoptysis [5]. In this case, on the third day of hospitalization, a fatal situation occurred when hypoxemia occurred due to blood influx into the unaffected lung. In the literature, there are reports of various means such as bronchial blockers [6], intrabronchial stents [7], bronchial fillers [8], argon plasma coagulation [9, as emergency hemostasis measures for massive hemoptysis, and all of these methods require specialized equipment and technical proficiency. Although separate lung ventilation was also prepared this time, it was difficult to maintain oxygenation during the procedure, and cardiac arrest was imminent, so V-V ECMO was introduced first.

V-V ECMO is effective in improving the mortality rate for severe respiratory failure [10]. However, the use of ECMO in palliative situations like this case is not generally indicated and requires careful discussion [11]. In this case, considering his clinical stage and age in his 60s, we decided to use ECMO. Antithrombotic drugs are not used in combination to prevent exacerbation of hemoptysis, and the policy was to respond by circuit replacement when intracircuit thrombus formation occurred. The use of ECMO is usually contraindicated for cases that anticoagulant therapy is difficult [12]; on the other hand, there is also a report that because of the complication of refractory bleeding when using ECMO, anticoagulant therapy was not administered, and blood products were replaced [13].

Radiotherapy has been reported to be useful for hemostasis [14], but there is also a report that it is not useful for massive hemoptysis in the acute phase [15]. Since the hemostatic effect of radiotherapy takes a long time to appear, it is necessary to use some emergency hemostatic means or, as in this case, use V-V ECMO and blood products to get through the period until the effect of radiotherapy appears. The effect of the prescribed dose on the hemostatic effect is not clear, and 30 Gy/10 fr, which was used in this case, is often used [16]. This time, the pleural effusion volume increased and decreased, and the position reproducibility of the target volume was poor, so the correction of the position error by IGRT was effective. To minimize the effect on the unaffected lung, we adopted parallel opposing fields by 3D-CRT.

Multidisciplinary treatment for lung cancer patients usually refers to a combination of surgery, drug therapy, and radiotherapy, as well as including the emergency medical team, will increase treatment options for massive hemoptysis and may lead to improved treatment outcomes.

## Conclusion

We experienced a case in which V-V ECMO and radiotherapy were effective in saving life and stopping bleeding in massive hemoptysis from lung cancer in which surgery and IVR were not indicated.

## Data Availability

Data sharing is not applicable to this article as no datasets were generated or analyzed during in the study.
